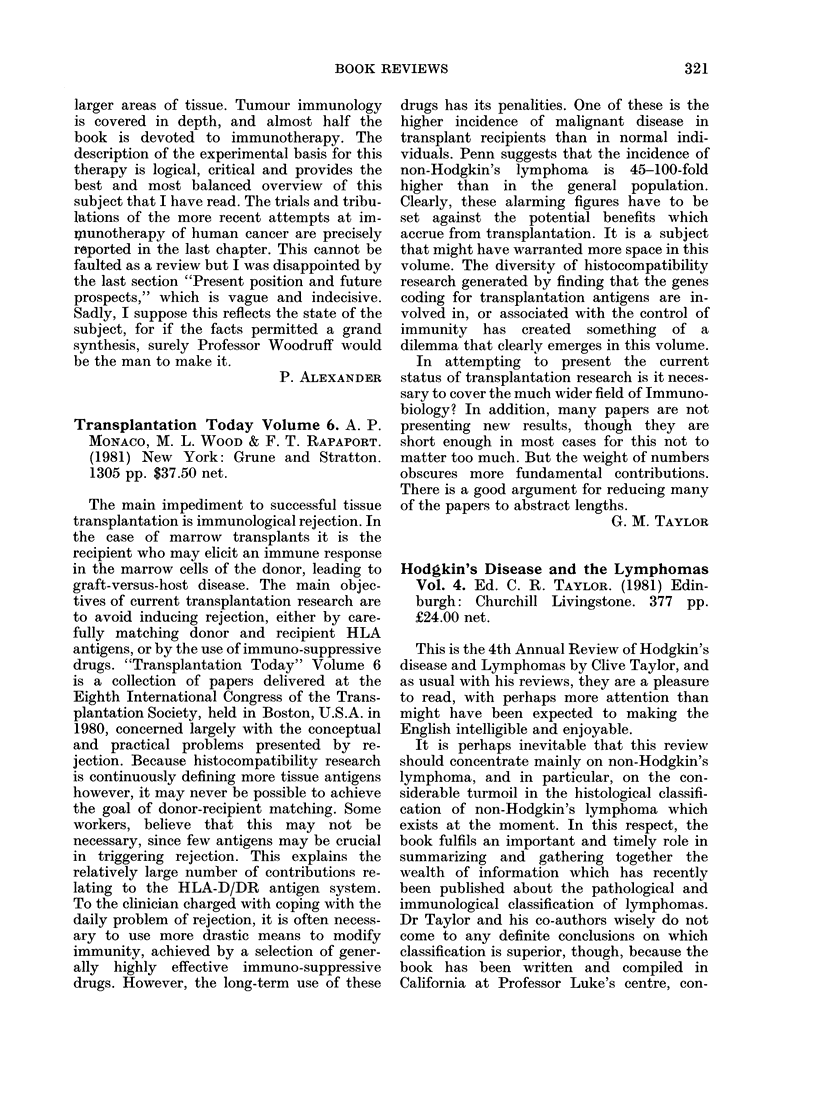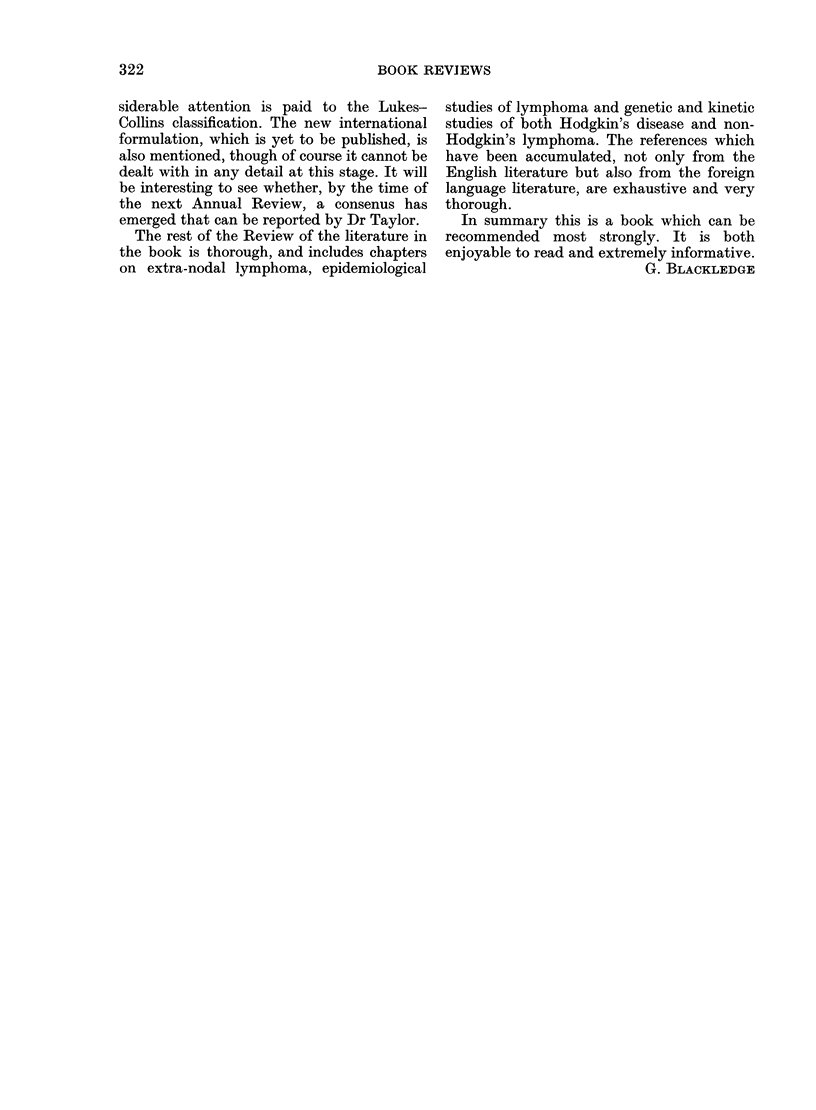# Hodgkin's Disease and the Lymphomas Vol. 4

**Published:** 1982-02

**Authors:** G. Blackledge


**Hodgkin's Disease and the Lymphomas Vol. 4.** Ed. C. R. TAYLOR. (1981) Edinburgh: Churchill Livingstone. 377 pp. £24.00 net.

This is the 4th Annual Review of Hodgkin's disease and Lymphomas by Clive Taylor, and as usual with his reviews, they are a pleasure to read, with perhaps more attention than might have been expected to making the English intelligible and enjoyable.

It is perhaps inevitable that this review should concentrate mainly on non-Hodgkin's lymphoma, and in particular, on the considerable turmoil in the histological classification of non-Hodgkin's lymphoma which exists at the moment. In this respect, the book fulfils an important and timely role in summarizing and gathering together the wealth of information which has recently been published about the pathological and immunological classification of lymphomas. Dr Taylor and his co-authors wisely do not come to any definite conclusions on which classification is superior, though, because the book has been written and compiled in California at Professor Luke's centre, considerable attention is paid to the Lukes–Collins classification. The new international formulation, which is yet to be published, is also mentioned, though of course it cannot be dealt with in any detail at this stage. It will be interesting to see whether, by the time of the next Annual Review, a consenus has emerged that can be reported by Dr Taylor.

The rest of the Review of the literature in the book is thorough, and includes chapters on extra-nodal lymphoma, epidemiological studies of lymphoma and genetic and kinetic studies of both Hodgkin's disease and non-Hodgkin's lymphoma. The references which have been accumulated, not only from the English literature but also from the foreign language literature, are exhaustive and very thorough.

In summary this is a book which can be recommended most strongly. It is both enjoyable to read and extremely informative.

G. Blackledge